# Estimation of process performance index for the two-parameter exponential distribution with measurement error

**DOI:** 10.1038/s41598-023-29393-3

**Published:** 2023-02-09

**Authors:** Yi Li, Jyun-You Chiang, Yajie Bai, Kuang-Cheng Chai

**Affiliations:** 1 School of Statistics, Southwestern University of Finance and Economics, Chengdu, 611130 China; 2 Business School, Guilin University of Electronic Technology, Guilin, China

**Keywords:** Statistics, Engineering

## Abstract

Measurement errors are inevitable in practice, but they are not considered in the existing process performance index. Therefore, we propose an estimation method of process performance index for the two-parameter exponential distribution with measurement errors to fill this gap. In this paper, the relationship between the unobservable actual value and measurement value is considered as full error model, and the maximum likelihood estimation method is considered to obtain the unknown parameters. In addition, we also use the Bootstrap method to construct confidence intervals of process performance index. The performance of the proposed estimation is investigated in terms of bias, mean square error (MSE) and average interval length. Simulation results show that the proposed estimator outperforms other estimators. Finally, an example of the mileage data of the military personnel carrier is given to illustrate the implementation of the proposed estimation method.

## Introduction

Process Capability Index (PCI) is a very important analytical tool to show the performance and potential capability of a process, so that producers and consumers can understand the quality of the process through the PCI. At present, there are many commonly used PCIs that can help us improve process quality, such as $C_{p}$, $C_{pk}$, $C_{pm}$, $C_{pmk}$ and so on. When we evaluate the product survival lifetime of process capability, it is clear that longer lifetime means better product quality and capability. Montgomery^1^ and Kane^2^ provided the process performance index $C_{L}$ for properly measuring the larger-the-better type quality characteristics. Recently, there are many studies on process performance index $C_{L}$, such as Yu et al.^3^, Wu et al.^4^, Tsai et al.^5^, and so on.

In the past studies on process capability analysis, most of the studies assumed that the process follows a normal distribution. However, in many applications, the observed data may follow a non-normal distribution such as two-parameter exponential distribution, such as the mileage to failure of a military personnel carrier (Lawless^6^), the high-voltage data of current in a P-type high-voltage metal oxide semiconductor transistor on a flash memory wafer (Kao^7^), the lifetime data of electronic products (Yang et al.^8^). In recent years, some scholars have also inferred the process capability analysis for the two-parameter exponential distribution, for example, Lee et al.^9^ proposed the lifetime performance index based on the type II right-censored sample. Wu et al.^10^ studied the maximum likelihood estimation (MLE) of the lifetime performance index based on the type II right-censored sample. Wu and Chiu^11^ proposed 14 estimators for estimating the lifetime performance index based on the multiply type II censored sample. Ahmadi et al.^12^ provided an estimation method for process performance index based on generalized order statistics.

When a process involves measurement, we obtain data with measurement errors due to the environment, people or measurement equipment, see van Leeuwen et al.^13^, Singh et al.^14^, Ge et al.^15^, and Yang et al.^16^. In terms of process capability estimation, Mittag^17^ considered the effect of measurement error on traditional PCIs. Bordignon and Scagliarini^18^ studied the bias and mean square error of measurement error on $C_{p}$ and $C_{pk}$. Pearn and Liao^19^ proposed the estimation and testing of one-sided PCIs with the presence of measurement errors. Baral and Anis^20^ applied the method of Generalized Confidence Interval to measure the process capability index $C_{pm}$ in presence of measurement errors. Sadeghpour Gildeh and Abbasi Ganji^21^ proposed the incapability index in presence of measurement error and investigates its statistical properties. Leony and Lin^22^ evaluated candidate processes based on quality loss using the incapability index. Afshari et al.^23^ provided the effects of classical and fuzzy estimation methods for multivariate process capability indices under a linear covariate error model. Although measurement error may hinder process quality improvement, research on process performance index with measurement error is very limited, and most studies assume that the data follow a normal distribution. In addition, the estimation of the process performance index does not consider the occurrence of measurement errors for the two-parameter exponential distribution. However, measurement errors may be unavoidable in practice. Therefore, we propose the estimation of process performance index for two-parameter exponential distribution with measurement error.

The remainder of this article is organized as follows. In section "The process performance index with additive measurement error model", the process performance index with full error model is introduced. When the parameters are unknown, the inference of the maximum likelihood estimation method is given in section "Parameter estimation". In section "Simulation study", the performance of the proposed estimation method is verified using the Monte Carlo simulation method. Later, an example is given to implement the proposed estimation method in section "Real data analysis". Finally, the main conclusions are summarized in section “Discussion”.

## The process performance index with additive measurement error model

Let $X$ be a random variables, which is taken from a two-parameter exponential distribution, denoted by $X \sim \exp (\theta ,\;\lambda )$. The probability density function (PDF) and cumulative distribution function (CDF) of the $\exp (\theta ,\;\lambda )$ are defined by

1 $$f\left( {x;\theta ,\lambda } \right) = \frac{1}{\lambda }e^{{ - \frac{x - \theta }{\lambda }}} , x > \theta \ge 0, \lambda > 0,$$

and

2 $$F\left( {x;\theta ,\;\lambda } \right) = 1 - e^{{ - \frac{x - \theta }{\lambda }}} ,\;x > \theta \ge 0,\;\lambda > 0,$$

respectively, where $\theta$ and $\lambda$ are the location (or threshold) parameter and scale parameter, respectively. The mean and standard deviation of the $\exp (\theta ,\;\lambda )$ are

3 $$\mu_{x} = \theta + \lambda ,$$

and

4 $$\sigma_{x} = \lambda ,$$

respectively. Montgomery^1^ and Kane^2^ developed a process performance index $C_{L}$ for properly measuring the larger-the-better type quality characteristics. $C_{L}$ is defined as follows:

5 $$C_{L} = \frac{\mu - L}{\delta },$$

where $\mu$ denotes the process mean, $\delta$ represents the process standard deviation, and $L$ is the lower specification limit. To assess the lifetime performance of variable $X$, form Eqs. (3) and (4), the process performance index of $X$ can be rewritten as:

6 $$C_{LX} = \frac{\theta + \lambda - L}{\lambda } = 1 - \frac{L - \theta }{\lambda }.$$

Although modern manufacturing processes are very advanced, there are generally measurement errors. Therefore, let $Y$ denote the measurement value and $X$ denote the unobservable actual value, the relationship between $Y$ and $X$ can be expressed as:

7 $$Y = A + BX + \varepsilon ,$$

where $A$ and $B$ are constants and $\varepsilon$ is the gauge precision error. In real applications, parameter $A$ is the measurement error caused by sensor setup/calibration bias or drift when using the sensor in a specific environment, and parameter $B$ is the measurement sensitivity. So, parameters $A$ and $B$ are dependent on the gauge location error. Suppose $X$ and $\varepsilon$ are stochastically independent, and $\varepsilon$ follows a normal distribution with mean 0 and variance $\sigma^{2}$. The PDF of $Y$ is obtained by (Li et al.^24^)

8 $$f\left( y \right) = \frac{1}{{\sqrt {2\pi } B\lambda \sigma }}e^{{\frac{\theta }{\lambda } + \frac{A}{B\lambda } - \frac{y}{B\lambda } + \frac{{\sigma^{2} }}{{2B^{2} \lambda^{2} }}}} \Phi \left( {\frac{{\left( {y - \frac{{\sigma^{2} }}{B\lambda }} \right) - (A + B\theta )}}{\sigma }} \right),$$

where $\Phi ( \cdot )$ is the CDF of the standard Normal distribution, and denoted by $Y \sim N2\exp \left( {\theta ,\;\lambda ,\;A,\;B,\;\sigma^{2} } \right)$. Since $X$ and $\varepsilon$ are stochastically independent, the mean and variance of $Y$ are as follows,

9 $$\mu_{y} = A + B(\theta + \lambda ),$$

and

10 $$\sigma_{y} = \sqrt {B^{2} \lambda^{2} + \sigma^{2} } ,$$

respectively. Therefore, the process performance index of $Y$ can be rewritten as:

11 $$C_{LY} = \frac{{A + B\left( {\theta + \lambda } \right) - L}}{{\sqrt {B^{2} \lambda^{2} + \sigma^{2} } }}.$$

In addition, we can also use Eqs. (6) and (11) to express the relationship between $C_{LX}$ and $C_{LY}$ as follows:

12 $$C_{LX} = \frac{{C_{LY} \sqrt {B^{2} \lambda^{2} + \sigma^{2} } - \left( {A + (B - 1)\left( {\theta + \lambda } \right)} \right)}}{\lambda }.$$

from Eq. (12), when $\sqrt {B^{2} + \frac{{\sigma^{2} }}{{\lambda^{2} }}} \left[ {1 - \frac{{A + \left( {B - 1} \right)\left( {\theta + \lambda } \right)}}{{A + B\left( {\theta + \lambda } \right) - L}}} \right] > 1$, then $C_{LX} > C_{LY}$. Conversely, when $\sqrt {B^{2} + \frac{{\sigma^{2} }}{{\lambda^{2} }}} \left[ {1 - \frac{{A + \left( {B - 1} \right)\left( {\theta + \lambda } \right)}}{{A + B\left( {\theta + \lambda } \right) - L}}} \right] < 1$, then $C_{LX} < C_{LY}$. Additionally, when $A = 0$ and $B = 1$, Eq. (7) reduces to the special case $Y = X + \varepsilon ,$ then the Eqs. (11) and (12) can be rewritten as:

13 $$C_{LY * } = \frac{{\left( {\theta + \lambda } \right) - L}}{{\sqrt {\lambda^{2} + \sigma^{2} } }},$$

and

14 $$C_{LX * } = \frac{{C_{LY * } \sqrt {\lambda^{2} + \sigma^{2} } }}{\lambda }.$$

And from Eq. (14), we can find that $C_{LX * } > C_{LY * }$ for $A = 0$ and $B = 1$.

## Parameter estimation

Let $X_{1} ,\;X_{2} , \ldots ,\;X_{n}$ and $Y_{1} ,\;Y_{2} , \ldots ,\;Y_{n}$ be two independent random samples, where $X \sim \exp (\theta ,\;\lambda )$ and $Y \sim N2\exp \left( {\theta ,\;\lambda ,\;A,\;B,\;\sigma^{2} } \right)$. In this paper, the parameters $A,B$ and $\sigma^{2}$ are assumed to be known, but in practice, the estimators of $A,B$ and $\sigma^{2}$ can be estimated using the least squares method. So we can use the maximum likelihood estimation method to estimate the parameters $\theta$ and $\lambda$. Based on a random sample $Y_{1} ,\;Y_{2} , \ldots ,\;Y_{n}$, the log-likelihood function can be presented

15 $$\begin{aligned} L\left( {\theta ,\;\lambda } \right) \propto & - nlog\left( \lambda \right) + \frac{Bn\theta + nA}{{B\lambda }} + \frac{{n\sigma^{2} }}{{2B^{2} \lambda^{2} }} - \frac{1}{B\lambda }\sum\limits_{i = 1}^{n} {y_{i} } \\ & + \sum\limits_{i = 1}^{n} l og\Phi \left( {\frac{{\left( {y_{i} - \frac{{\sigma^{2} }}{B\lambda }} \right) - (A + B\theta )}}{\sigma }} \right). \\ \end{aligned}$$

The first-order derivatives of $L\left( {\theta ,\;\lambda } \right)$ are obtained as follows:

16 $$\frac{\partial L(\theta ,\;\lambda )}{{\partial \theta }} = \frac{n}{\lambda } - \frac{B}{\sigma }\sum\limits_{i = 1}^{n} {\varphi ,}$$

and

17 $$\frac{\partial L(\theta ,\;\lambda )}{{\partial \lambda }} = n + \frac{n\theta }{\lambda } + \frac{{n\sigma^{2} }}{{B^{2} \lambda^{2} }} - \frac{{\sum\limits_{i = 1}^{n} {y_{i} + } \sigma \sum\limits_{i = 1}^{n} \varphi - nA}}{B\lambda },$$

where $\varphi = \phi \left( {\frac{{\left( {y_{i} - \frac{{\sigma^{2} }}{B\lambda }} \right) - (A + B\theta )}}{\sigma }} \right)/\Phi \left( {\frac{{\left( {y_{i} - \frac{{\sigma^{2} }}{B\lambda }} \right) - (A + B\theta )}}{\sigma }} \right)$, and $\phi ( \cdot )$ is the PDF of the standard Normal distribution. The MLE of the parameter $(\theta ,\lambda )$, denoted by $(\hat{\theta },\hat{\lambda })$, by solving the likelihood equations, which are from setting Eqs. (16) and (17) to be zero. Replacing $(\theta ,\lambda )$ with $(\hat{\theta },\hat{\lambda })$ in Eq. (12), the MLE of the $C_{LX}$ is given by

18 $$\hat{C}_{LX} = \frac{{\hat{C}_{LY} \sqrt {B^{2} \hat{\lambda }^{2} + \sigma^{2} } - \left( {A + (B - 1)\left( {\hat{\theta } + \hat{\lambda }} \right)} \right)}}{{\hat{\lambda }}},$$

where $\hat{C}_{LY} = (A + B\left( {\hat{\theta } + \hat{\lambda }} \right) - L)/\sqrt {B^{2} \hat{\lambda }^{2} + \sigma^{2} } .$ Since the sampling distribution of $\hat{C}_{LX}$ is difficult to obtain, the parametric Bootstrap method is considered to obtain the confidence interval of $C_{LX}$. The principle of the parametric Bootstrap method is to use repeated sampling to obtain the empirical distribution of $\hat{C}_{LX}$, and then use the empirical distribution to obtain the confidence interval. For the discussion of the parametric Bootstrap method, please refer to DiCiccio and Efron^25^, Wang et al.^26^ and Paradis et al.^27^. Therefore, the steps to obtain the confidence interval of $C_{LX}$ are as follows:

$Step 1:$ Using Eq. (16) and (17), the MLEs $\hat{\theta }$ and $\hat{\lambda }$ can be computed based on $n$ samples, $Y_{1} ,\;Y_{2} , \ldots ,\;Y_{n}$.

$Step 2:$ Given $A,B$ and $\sigma^{2}$, generate parametric bootstrap samples $y_{1}^{ * } ,y_{2}^{ * } , \ldots y_{n}^{ * }$ from $N2{\exp} \left( {\hat{\theta },\;\hat{\lambda },\;A,\;B,\;\sigma^{2} } \right).$

$Step 3:$ Find the MLEs of $\theta$ and $\lambda$, using $y_{1}^{ * } ,y_{2}^{ * } , \ldots y_{n}^{ * }$ and denote the reproduced MLEs by $\hat{\theta }^{ * }$ and $\hat{\lambda }^{ * }$, respectively.

$Step 4:$ Compute the bootstrap estimate of the $C_{LX}$ according to the Eq. (18), and denoted by $\hat{C}_{LX}^{ * }$.

$Step 5:$ Repeat $Step 2$ to $Step 4$ $N$ times, where $N$ is a given large positive integer. Then, arrange all the bootstrap estimates in ascending order to obtain the ordered bootstrap estimates as $\hat{C}_{LX,\;1}^{ * } < \hat{C}_{LX,\;2}^{ * } < \cdots < \hat{C}_{LX,\;N}^{ * }$.

$Step 6:$ Given an significant level $p$, and the $100 \times \left( {1 - p} \right)\%$ confidence interval of $C_{LX}$ is the $\left( {p/2} \right)th$ to $\left( {1 - p/2} \right)th$ empirical quantiles of the bootstrap sample, $\hat{C}_{LX,\;1}^{ * } ,\;\hat{C}_{LX,\;2}^{ * } , \ldots ,\;\hat{C}_{LX,\;N}^{ * }$.

## Simulation study

In this section, the performance of the proposed estimate $\hat{C}_{LX}$ is investigated by Monte Carlo simulations, the performance of proposed estimation method are investigated in terms of simulated absolute bias (${\text{|Bias|}}$) and mean square errors (MSEs), respectively, and are defined as follows:

$${\text{|Bias|}} = \left| {\frac{1}{10000}\sum\limits_{i = 1}^{10000} {\left( {\hat{C}_{L} - C_{LX} } \right)} } \right|,$$

and

$${\text{MSE}} = \frac{1}{10000}\sum\limits_{i = 1}^{10000} {\left( {\hat{C}_{L} - C_{LX} } \right)^{2} ,}$$

where $\hat{C}_{L}$ is the estimator of $C_{LX}$.

**Figure 1:**
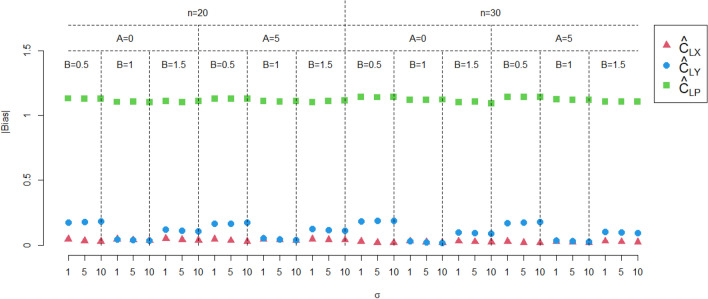
The absolute biases of the three estimators.

**Figure 2:**
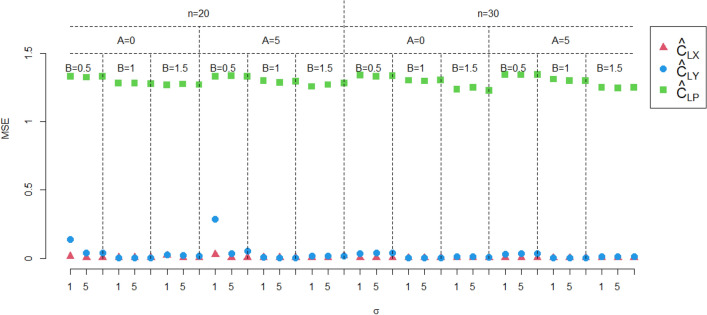
The MSEs of the three estimators.

In this section, we consider comparing the performance of the proposed estimator $\hat{C}_{LX}$ with $\hat{C}_{LY}$ and $\hat{C}_{LP}$, where $\hat{C}_{LY}$ is the estimator of $Y$, and $\hat{C}_{LP}$ is the estimator proposed by Zhu et al.^28^. In this study, the two-parameter exponential distribution with $\theta = 151.85$ and $\lambda = 835.39$, which is tied with the real application in section "Real data analysis", is concerned. The rest inputs for the simulation study are sample size $n = 20,30$, intercept $A = 0,5$, slope $B = 0.5,1,1.5$, standard deviation of error term $\sigma = 1,\;5,\;10$, lower lifetime limit $L = 171.0144$. Then, the bias and MSEs are calculated through 10,000 simulation runs. All simulation results are shown in Figs. 1 and 2. From Figs. 1 and 2, we have the following findings,

- The performance of $\hat{C}_{LX}$ outperforms $\hat{C}_{LY}$ and $\hat{C}_{LP}$.
- As the sample size increases, both the bias and MSE of $\hat{C}_{LX}$ decrease.
- Changes in the intercept $A$ do not affect the performance of $\hat{C}_{LX}$. But when $A$ increases, both the bias and MSE of $\hat{C}_{LY}$ decrease.
- As the slope $B$ increases, both the bias and MSE of $\hat{C}_{LX}$ increases.
- When $\sigma$ increases, both the bias and MSE of $\hat{C}_{LX}$ decrease.

In summary, when measurement error is present, using a process capability estimator without measurement error will produce misleading results, especially when $A \ne 0$ and $B \ne 1$.

To evaluate the performance of the proposed estimation method for the confidence interval of $C_{LX}$. The average interval length (denoted by Length) based on 10,000 simulation runs are used to evaluate confidence intervals. The parameters we consider are set as follows, $\theta = 151.85$, $\lambda = 835.39$, $A = 0$, $B = 1,1.5$, $\sigma = 1,\;5,\;10$, significant level $p = 0.05$ and sample size $n = \, 20,30$. The confidence interval for bootstrap method is evaluated based on $N = 5,000$ bootstrap samples. Table 1 exhibits the simulated Length for the proposed estimation. This result is consistent with the theory of Bootstrap method, that is, as the sample size increases, the average length of the interval will decrease.

**Table 1:** The average interval length of proposed confidence interval for $C_{LX}$.

$n = 20$			$n = 30$		
$B$	$\sigma$	Length	$B$	$\sigma$	Length
1	1	0.220	1	1	0.144
	5	0.221		5	0.146
	10	0.226		10	0.151
1.5	1	0.220	1.5	1	0.144
	5	0.221		5	0.145
	10	0.223		10	0.148

## Real data analysis

An example regarding the mileage to failure of a military personnel carrier to illustrate the application of the proposed method. The dataset, containing the mileages before failure for each of the 19 military personnel carriers, was reported in Lawless^6^ and also discussed by Lee et al.^9^. Lee et al.^9^ mentioned that the data follow a two-parameter exponential distribution. So in this example, we assume that this data is the actual mileage that cannot be observed, and denoted by $X$. The observable mileage $Y$ may be affected by a problem with the odometer sensor, or the sensor may be too far from the induction coil (ring gear) in the transmission, causing the observed mileage to differ from the actual mileage. Assuming that $A = 0$, $B = 1$ and $\sigma = 5$ are known, we randomly generate observable data $Y$ as

167 197 280 320 395 497 537 630 705 779

882 1010 1102 1186 1458 1606 1992 2361 2872.

Data *Y* can be downloaded in supplementary file. According to the data $Y$, calculated MLEs of $\theta$ and $\lambda$ from Eqs. (16) and (17) are $\hat{\theta } = 151.85$ and $\hat{\lambda } = 835.39$, respectively. The lower lifetime (i.e., mileage) limit $L$ is assumed to be 171.0144, the MLE of the $C_{LX}$ can be obtained from Eq. (18) and is equal to $\hat{C}_{LX} = 0.977$, and the confidence interval for $C_{LX}$ is $\left( { - \;0.410, \, 0.99} \right)$. So we can also estimate the failure rate of process to be $p\left( {X < L} \right) = 1 - e^{{ - \frac{{L - \hat{\theta }}}{{\hat{\lambda }}}}} = 0.02268$. In other words, 22,680 out of every million personnel carriers are substandard. In addition, in the paper of Lee et al.^9^, they also provided parameter estimators for $\theta$, $\lambda$ and $C_{LX}$ to be 115.60, 881.61 and 0.94, respectively, based on actual mileage data $X$. These results are not much different from that of the proposed estimation in this paper, which also shows that the proposed estimation method is reliable for this data. It is worth noting that $Y$ does not follow a two-parameter exponential distribution, so we cannot use the estimation method proposed by Lee et al.^9^ for the data $Y$.

## Discussion

Measurement errors are inevitable in the measurement processes due to environment, measurement equipment or personnel, so estimated process performance index can lead to misleading conclusions if measurement errors are not taken into account. Therefore, we propose a method for estimating process performance index for a two-parameter exponential distribution with measurement error. During the estimation of process performance index $C_{L}$, the measurement error model considered in this paper is a full error model $Y = A + BX + \varepsilon ,$ which can be reduced to model $Y = X + \varepsilon$ or model $Y = X$. Therefore, the estimation methods of Wu et al.^10^, Tong et al.^29^ and Lee et al.^9^ for the complete data are a special case of the proposed estimation method. We estimate the unknown parameters using the maximum likelihood method, and the Bootstrap method is considered to construct confidence interval of $C_{LX}$. In the results of the simulation study we can find that the proposed estimation method outperforms the rivals. In addition, the process performance index is estimated by taking the mileage of a military personnel carrier as an example. It is shown that the estimation results using the proposed estimation method in the data with measurement errors are reliable (Supplementary file).

The relationship between the unobservable actual value and measurement value considered in this paper is a linear relationship, without considering other possible relational expressions, and the real observation must follow a two-parameter exponential distribution. Therefore, other possible relational expressions and consideration of different data distributions are potential directions for future research.

## Supplementary Information

Supplementary Information.

## Data Availability

All data generated or analysed during this study are included in this published article.
